# Effect of Human Placental Extract on Health Status in Elderly Koreans

**DOI:** 10.1155/2012/732915

**Published:** 2012-02-20

**Authors:** Mihee Kong, Sat Byul Park

**Affiliations:** ^1^Department of Family Medicine, School of Medicine, Jeju National University, Jeju 690-756, Republic of Korea; ^2^Department of Family Practice and Community Health, School of Medicine, Ajou University, Suwon 443-721, Republic of Korea

## Abstract

*Objectives*. Human placental extract (HPE) has begun to be used in Korea in various ways to improve health, even though evidence-based data is insufficient. This study investigated the effects of HPE on health status in elderly Koreans. *Design*. Randomized, single-blind, and case-control study design. *Setting and Participants*. Thirty-nine community-dwelling healthy Koreans ≥65 years of age. *Intervention*. The participants were randomly categorized into a placebo group (*n* = 17) and HPE group (*n* = 22). The HPE group received abdominal subcutaneous injections of HPE for 8 weeks. The placebo group was injected with normal saline. *Measurements*. The degree of health status was surveyed by the Korean health status measure for the elderly (KoHSME V1.0) at baseline and the end of the study. *Results*. In the HPE group, the scores of physical function, sexual life, and general heath perception at the end of the study period were significantly improved from baseline (*P* = .007, .020, and .005, resp.), while the health status of the placebo group remained unchanged during the study period. There was a significant difference over the study period between the two groups in the mean change of the physical function score (*P* = .036). *Conclusion*. A HPE injection regimen can improve the health status in elderly Koreans.

## 1. Introduction

Human placenta has been a historical folk remedy in Oriental countries. In Japan, the hydrolysate of human placenta, which contains therapeutic compounds, has long been refined as human placental extract (HPE). In Korea, HPE has been approved for the improvement of liver function and menopausal symptoms since its importation from Japan in 2003. In Korea, HPE has been approved and is now widely used for improvement of fatigue, skin whitening, and antiaging. Despite this popularity, the efficacy of HPE has not been sufficiently researched.

Studies using animal models have provided evidence for liver function improvement and skin whitening attributes of HPE by liver regeneration and antimelanocyte activity [1–5]. Improvements of menopausal symptoms and fatigue have been indicated in several studies, including one from our group [6, 7].

With the aim of investigating HPE in more rigorous detail, the present randomized controlled trial was undertaken to determine the influence of HPE on the health status in elderly Koreans.

## 2. Methods

### 2.1. Study Approval

This study was approved by the Institutional Review Board of Ajou University Hospital, Suwon, Republic of Korea (AJIRB-CRO-05-211).

### 2.2. Participants

A public notice posted on the notice board of Ajou University Hospital between May and July, 2006, recruited research participants. Details of the study were explained to potential volunteers prior to obtaining informed consent.

Initial screening included past/current medical history and a physical examination. Eligible participants were Koreans ≥65 years of age. Subjects with history of chronic renal disease were excluded if their plasma creatinine levels exceeded 1.5 mg/dL, if they had history of liver cirrhosis or other hemmorhogic diseases, history of any cancer, or history of taking medications such as thrombolytic agents. Forty-two subjects (20 males, 22 females) were selected. The sample size calculations were based on the assumptions of a standardized effect size of 0.90, statistical power of 95%, and a (1-sided) type I error 5%. To meet these assumptions, 42 patients were required.

### 2.3. HPE Preparation and Study Design

The hydrolysate of human placenta (HPE; Laennec; Green Cross Japan Bio Products, Yongin, Korea) has been previously described [6]. It is an aqueous extract of human placenta that is approved by the Korea Food and Drug Administration for sale and use in the treatment of chronic liver diseases. To prepare the product, human placenta collected at the time of full-term delivery was immediately placed in ice. Each placenta is tested to ensure the absence of human immunodeficiency virus, hepatitis B virus, and hepatitis C virus. The acceptable placentas are cut into pieces and extracted with water through enzymatic molecular separation and chemical hydrolysis. Hydrolysis utilizes hydrochloric acid and heating for 15–17 hours. At the final process, the extract is sterilized by steam at 121°C for 30 minutes and sealed in ampoules for sale.

The subjects were randomly assigned to receive subcutaneous injection of either placebo (normal saline, *n* = 18) or HPE (*n* = 22) for 8 weeks. Participants were injected with the appropriate preparation in the following schedule: 4 mL twice weekly for the first 2 weeks, 2 mL twice a week for the next 2 weeks, and 2 mL once weekly for the last 4 weeks. Each participant visited the hospital 12 times during the 8-week study for the injection, and the total amount injected was 32 mL. All subjects had their eyes covered during injection to blind them to the identity of the injected preparation. Health status was assessed by a questionnaire and metabolic parameters including cardiovascular risk factors that were measured at baseline and after 8 weeks.

### 2.4. Questionnaire for Assessment of Health Status

The survey questionnaire for Korean Health Status Measure for the Elderly (KoHSME V1.0) [8] was used. KoHSME consists of a list of 44 items, which each has a 1–5 Likert response scale, except for pain intensity, which uses a visual analogue scale. The composite score is the sum of six dimension scores: physical function (19 items), emotional function (13 items), social function (6 items), sexual function (1 item), general health perception (3 items), and bodily pain (2 items). Four dimension scores (physical function, emotional function, general health perception, and bodily pain) have subscales. The physical function score includes daily activity, fitness, and aging physiological function subscales. The emotional function score includes depression and anxiety subscales. The general health perception score includes health status perception, health status change, and functional health satisfaction subscales. The bodily pain includes pain frequency and intensity subscales. The internal correlation reliability of physical function was 0.94, emotional function was 0.90, and social function was 0.90 at the time of development of this questionnaire.

### 2.5. Metabolic Parameters

Body measurement and metabolic parameters were similar to those of our prior study [6], including body mass index (BMI), blood pressure, hemoglobin, platelet count, white blood cell count, glucose, aspartate aminotransferase (AST), alanine aminotransferase (ALT), and gamma glutamyl transpeptidase (*γ*-GTP). Metabolic parameters were measured exactly as described previously [6]. Insulin-like growth factor 1 (IGF-1) was also measured using an IRMA kit (Diagnostic Systems Laboratories, Webster, TX, USA).

### 2.6. Statistical Analyses

Statistical analyses utilized SPSS for windows, version 11.5 (SPSS, Chicago, IL, USA). A nonparametric test was used to compare HPE with placebo at baseline and the end of the study (continuous variables by Mann-Whitney test, categorical variables by Pearson Chi-square or Fisher's Exact test). Wilcoxon-signed rank test was used to assess the change in each group at the beginning and end of the study period. All values are shown as mean ± standard deviation (SD). A *P* value <0.05 indicated statistical significance.

## 3. Results

One subject in the placebo group did not complete the study, withdrawing for a personal reason. Thirty-nine participants completed the study (22 in the HPE group and 17 in the placebo group).

### 3.1. Clinical Characteristics

There were no significant differences between the HPE and placebo groups at baseline concerning gender, age, BMI, prevalence/nature of cardiovascular diseases, use of antihypertensive medication, use of oral hypoglycemic agents for diabetes, and use of medication for hyperlipidemia (Table 1). Laboratory test results (Table 1) were also similar between the two groups.

### 3.2. Changes in Health Status

All mean KoHSME scores did not differ significantly between the HPE and placebo groups at baseline. In the HPE group, the mean scores of physical function, sexual function, and general health perception at the end of the study period were significantly improved from those at baseline (Table 2). Although the mean scores of emotional function and bodily pain were not different at baseline and the end of the study period, the depression score with subscale of the emotional function and pain frequency score with subscale of the pain at the end of the study period were significantly improved, compared to baseline (Table 2). However, all health status scores in the placebo group were not significantly different at baseline and the end of the study. There was a significant difference between the two groups in the mean change of physical function score during the study period (*P* = 0.036, Table 2). In particular, the aging physiological function score with subscale of the physical function differed significantly through the study (*P* < 0.001, Table 2).

### 3.3. Changes of Risk Factors of Cardiovascular Disease

There were no significant changes in the risk factors of cardiovascular disease in the two groups during the study period, including blood pressure, fasting plasma glucose, insulin, insulin resistance index (HOMA-IR, QUICKI), lipid levels (total cholesterol, triglyceride, high-density lipoprotein cholesterol), and high sensitivity C-reactive protein level (Table 3).

## 4. Discussion

The proportion of the elderly in Korean society and globally is increasing rapidly as advances in medical science permit a longer life. With an extended life span, the quality of life and health status of the elderly, especially concerning depression and physical function, have become very important issues. Depression in elderly people is an important cause of weight loss, cognitive dysfunction, and death [9–11].

In the present study, health status improved in elderly Koreans (≥65-years-of-age) when they received an 8-week regimen of HPE injections. Improvements noted in general physical function in accord with the improved physiologic function and depressive mood were improved at the end of the study period, while these aspects were unchanged in the placebo group. Aging has been linked to increased inflammation [12]. The demonstrated anti-inflammatory activity of HPE [13, 14] suggests that HPE has potential in modulating age-related inflammation, with consequent health benefits. Furthermore, HPE is a rich resource of various bioactive substances such as RNA, DNA, peptides, aminoacids, proteins, lipids, and enzymes [15, 16]. The anabolic effect of these components assists energy production, which may improve age-related physical functions. Improved physical function could lessen the development of depression in the elderly.

The present study also assessed the influence of HPE on sexual functioning. Although IGF-1 was not changed, the noted improvement in sexual function may have reflected the general physiologic function improvement mediated by HPE. HPE displays anti-inflammatory activity that is the same as diclofenac [13]. The lessened pain frequency noted in participants receiving HPE can be explained by the anti-inflammatory effect of HPE. Lessened pain would be anticipated to lessen a depressive mood.

The current study is limited by the small number of participants, the use of only Koreans, and the lack of a long-term assessment. Further studies must address these shortcomings before the significance of the present findings can be verified. Also, the effectiveness of HPE treatment needs to be explored further in a global approach, in terms of different cultures and habits. Despite the limitations, the study is notable for the demonstration of improved health status in elderly Koreans who received HPE for 8 weeks.

## Figures and Tables

**Table 1:** Comparison of clinical and biochemical characteristics between the two study groups at baseline.

	Placebo (*n* = 17)	HPE (*n* = 22)	*P* value
Male patient, no. (%)	9 (52.9)	10 (45.5)	0.643
DM, no. (%)	2 (11.8)	4 (18.2)	0.679*
HTN, no. (%)	7 (41.2)	14 (63.6)	0.163
Hyperlipidemia, no. (%)	3 (17.6)	3 (13.6)	1.000*
Age (years)	69.1 ± 3.5	68.0 ± 2.4	0.353
Weight (kg)	65.6 ± 7.3	65.9 ± 10.6^a^	0.907
BMI (kg/m^2^)	25.2 ± 2.6	25.8 ± 3.4^a^	0.780
Hemoglobin (g/dL)	13.9 ± 1.1	14.3 ± 1.1	0.444
Platelets (×10^3^/*μ*L)	236.2 ± 51.6	247.4 ± 79.3	0.734
WBC (×10^3^/*μ*L)	7.139 ± 1.776	6.505 ± 1.449	0.436
Creatinine (mg/dL)	0.9 ± 0.2	0.9 ± 0.2	0.697
AST (U/L)	20.6 ± 5.2	22.2 ± 10.7	0.921
ALT (U/L)	20.1 ± 7.6	23.1 ± 16.1	0.798
*γ*-GTP (U/L)	19.7 ± 9.3	22.5 ± 7.8	0.152
IGF-1 (*μ*IU/mL)	180.4 ± 82.0	197.6 ± 86.3	0.444

Mean ± SD, ^a^one data point missing, HPE: human placental extract, DM: diabetes, HTN: hypertension, BMI: body mass index, WBC: white blood cell count, AST: asparate aminotransferase, ALT: alanine aminotransferase, *γ*-GTP: gamma glutamyl transpeptidase, IGF-1: Insulin-like growth factor-1, and P: group difference of continuous variable using Mann-Whitney test and categorical variable by Pearson Chi-square test, *by Fisher's Exact test (d/t cells have expected count <5).

**Table 2:** Change of KoHSME in the two study groups.

	Placebo (*n* = 17)			HPE (*n* = 22)			*P * ^‡^ value (Placebo versus HPE)^‡^
	Baseline	End	*P* ^†^	Baseline	End	*P* ^†^	
Physical function	3.6 ± 0.7^c^	3.5 ± 0.7^b^	0.625	3.5 ± 0.5^a^	3.8 ± 0.5^a^	0.007	0.036
Daily activity	4.1 ± 0.6^c^	3.9 ± 0.7^b^	0.420	4.2 ± 0.5^a^	4.1 ± 0.7^a^	0.983	0.657
Fitness	3.5 ± 0.9^b^	3.6 ± 0.7^a^	0.316	3.4 ± 0.7	3.6 ± 0.6	0.111	0.493
Aging physiological function	3.2 ± 0.8^a^	3.0 ± 0.8^a^	0.116	3.0 ± 0.6	3.7 ± 0.5	0.001	<0.001
Emotional function	3.7 ± 0.6^a^	3.7 ± 0.6^b^	0.690	3.6 ± 0.7	3.8 ± 0.6^a^	0.067	0.440
Depression	3.9 ± 0.7^a^	3.9 ± 0.7^b^	0.569	3.6 ± 0.8	3.9 ± 0.7^a^	0.025	0.479
Anxiety	3.4 ± 0.7^a^	3.3 ± 0.6^b^	0.727	3.5 ± 0.6	3.7 ± 0.7	0.364	0.493
Social function	3.6 ± 1.2^a^	3.7 ± 0.8^a^	0.699	3.7 ± 0.7	3.7 ± 0.7	0.881	0.858
Sexual function	2.5 ± 1.2^a^	3.0 ± 1.3^b^	0.230	2.4 ± 1.1	3.0 ± 1.1^a^	0.020	0.620
General health perception	2.7 ± 0.6^a^	2.5 ± 0.5^a^	0.254	2.9 ± 0.5	2.6 ± 0.6^a^	0.005	0.618
Health status perception	2.8 ± 0.9^a^	2.6 ± 0.9^a^	0.470	2.8 ± 0.7	2.9 ± 0.7	0.763	0.477
Health status change	2.8 ± 0.6^a^	2.4 ± 0.7^a^	0.083	3.0 ± 0.6	2.3 ± 0.6	0.002	0.218
Functional health satisfaction	2.6 ± 0.9^a^	2.5 ± 1.1^a^	0.717	3.0 ± 0.7	2.5 ± 0.9^a^	0.004	0.338
Bodily pain	2.7 ± 0.9^a^	3.4 ± 0.9^b^	0.149	2.9 ± 0.9	3.4 ± 0.8^b^	0.053	0.892
Pain frequency	2.4 ± 1.3^a^	3.2 ± 1.2^a^	0.067	2.6 ± 1.2	3.3 ± 0.6	0.043	0.843
Pain intensity	53.4 ± 21.5^a^	61.7 ± 18.5^b^	0.560	58.2 ± 19.2	59.5 ± 20.9^b^	0.569	0.812

Mean ± SD, ^a^1 data missing, ^b^2 data missing, ^c^: 3 data missing, ^†^change between end and baseline in each group (by Wilcoxon signed ranks test), ^‡^comparison of mean change between HPE and placebo groups (by Mann-Whitney test), KoHSME; Korean Health Status Measure for the Elderly, and HPE: human placental extract.

**Table 3:** Change of risk factors of cardiovascular disease in the two study groups.

	Placebo			HPE			*P* value (Placebo versus HPE)^‡^
	Baseline (*n* = 18)	End (*n* = 17)	Difference^†^	Baseline (*n* = 22)	End (*N* = 22)	Difference^†^	
SBP (mmHg)	132.1 ± 11.9^a^	129.6 ± 15.1^c^	−0.8 ± 16.4^c^	132.8 ± 11.24^d^	135.5 ± 13.0^b^	2.5 ± 11.3^bd^	0.860
DBP (mmHg)	81.5 ± 6.1^a^	85.0 ± 8.3^c^	4.6 ± 10.5^c^	83.9 ± 8.9	83.7 ± 9.0^b^	0.5 ± 9.6^b^	0.250
FPG (mg/dL)	101.8 ± 11.5	100.1 ± 9.7^d^	−1.9 ± 6.8^d^	102.5 ± 13.32^e^	97.0 ± 11.6^f^	−1.0 ± 9.3^f^	0.842
Insulin (*μ*IU/mL)	6.4 ± 3.7	5.0 ± 3.7	−1.5 ± 3.8	7.9 ± 3.8^d^	7.3 ± 5.0^d^	−1.2 ± 4.3	0.640
HOMA-IR	1.62 ± 1.02	1.13 ± 0.77	−0.51 ± 0.93	1.95 ± 1.06^d^	1.90 ± 1.42^d^	−0.05 ± 0.85^d^	0.094
QUICKI	0.37 ± 0.04	0.40 ± 0.06	0.03 ± 0.04	0.35 ± 0.05	0.36 ± 0.05	0.01 ± 0.03	0.062
Total cholesterol (mg/dL)	186.0 ± 34.0	184.8 ± 35.0	−2.1 ± 22.5	197.7 ± 36.9	195.4 ± 46.7	−2.3 ± 25.0	0.843
Triglyceride (mg/dL)	149.9 ± 64.3	144.6 ± 47.8	−8.4 ± 55.1	167.1 ± 83.3	143.3 ± 57.9^d^	−5.0 ± 61.2	0.966
HDL cholesterol (mg/dL)	51.8 ± 10.5	50.8 ± 5.9	−0.8 ± 7.4	49.9 ± 9.0	50.2 ± 7.5	1.7 ± 7.1^d^	0.480
hs-CRP (mg/dL)	0.12 ± 0.11^d^	0.08 ± 0.05^e^	−0.36 ± 1.35^e^	0.15 ± 0.15^d^	0.17 ± 0.19^d^	0.002 ± 0.105 ^g^	0.731

Mean ± SD, ^a^1 data missing, ^b^3 data missing, ^c^5 data missing, ^d^1 data exclusion d/t extreme value within group, ^e^2 data exclusion d/t extreme values within group, ^f^3 data exclusion d/t extreme values within group, ^g^4 data exclusion d/t extreme values within group, ^†^change from baseline (after-baseline), ^‡^P values are for the comparison between groups (change from baseline), HPE: human placental extract, SBP: systolic blood pressure, DBP: diastolic blood pressure, FPG: fasting plasma glucose, HOMA-IR: homeostasis model assessment of insulin resistance, QUICKI: the quantitative insulin sensitivity check index, HDL: high-density lipoprotein, and hs-CRP: high-sensitivity C reactive protein.
